# Second‐ and third‐year medical students’ clinical encounters in the emergency department

**DOI:** 10.1002/aet2.10937

**Published:** 2024-02-21

**Authors:** Ines Hoxha, Daniel J. Hekman, Benjamin Schnapp

**Affiliations:** ^1^ University of Wisconsin–Madison School of Medicine and Public Health Madison Wisconsin USA; ^2^ Department of Emergency Medicine University of Wisconsin–Madison School of Medicine and Public Health Madison Wisconsin USA

**Keywords:** curriculum, emergency medicine, students, medical

## Abstract

**Background:**

Experiential learning theory suggests that direct clinical experiences facilitate learning. Previous literature has focused primarily on the experiences of fourth‐year medical students. As more students gain early clinical exposure, it is important to understand the types of patients seen by junior students.

**Objectives:**

This study aims to categorize the clinical experiences of early (M2 and M3) students in the emergency department (ED).

**Methods:**

A retrospective review of the electronic health record of patients seen by M2s and M3s on a 2‐week emergency medicine rotation at a single urban academic ED in the Midwest was performed. Data elements extracted included total number of patients seen, Emergency Severity Index (ESI), disposition, and chief complaint. Students were not mandated to see any particular patients.

**Results:**

Medical students (248) saw 2994 total patients from 2018 to 2022. The median number of patients seen by each student was 12.0 (range 1–32). Pediatric patients made up 6.5% (*n* = 194) of total patients. Encounters were primarily ESI 2 or 3, which accounted for 89.4% of all patients (*n* = 2676). The most encountered complaints were abdominal pain, chest pain, and dyspnea, making up 15.6% (*n* = 467), 8.7% (*n* = 260), and 5.5% (*n* = 165), respectively, of total cases. Obstetrics/gynecology, hematologic, and environmental disorders were the least frequently encountered domains. No students saw all Clerkship Directors in Emergency Medicine (CDEM)–recommended complaints.

**Conclusions:**

There is significant variability in the ED encounters of M2s and M3s, with wide ranges of patient volume and presentations. This study provides some evidence that early students may not be meeting CDEM recommendations.

## INTRODUCTION

Medical schools are moving toward the earlier introduction of clinical rotations: 35% of medical schools end their preclerkship phase after 1 or 1.5 years.^1^ The earlier integration of clinical experiences is supported by experiential learning theory.

However, experiences during clinical rotations, including emergency medicine (EM), vary greatly across students.^2^, ^3^, ^4^, ^5^, ^6^, ^7^, ^8^ The differences in medical student EM experiences could explain the gaps in knowledge and residency preparation seen by program directors.^9^ Furthermore, students who are required to seek out a core set of clinical presentations perform better on standardized examinations.^10^

Most of the previous literature has focused on categorizing experiences of fourth‐year students on their EM clerkship.^11^ While a few studies have considered clinical experiences during the third year,^3^, ^12^, ^13^ these studies are limited and no studies, to our knowledge, have described the experiences of second‐year students. Given the importance of clinical exposure to knowledge building, the experiences of these junior students may be even more foundational. The aim of this study was to evaluate the clinical experiences of early medical students in the emergency department (ED).

## METHODS

### Study design and setting

This was a retrospective review of patients seen by medical students at a single urban academic Level I trauma ED in the Midwestern United States with approximately 60,000 visits per year. The curriculum is divided into three “phases.” In January of their second year, students begin Phase 2—a year of core clinical rotations consisting of 12‐week thematic blocks.^14^ During this year, students complete a 2‐week‐long required EM rotation, composed of five 8‐h ED shifts. Students were either in the second half of their M2 or in the first half of their M3 year during this time. Students were not mandated to see any particular patients; however, the EM experience is primarily adult‐focused. Supervising physicians are instructed to direct students to sign up and complete a note for patients they see. Physicians are told that notes from M2s can be used for billing, to encourage students to write notes.

### Data retrieval

Data were obtained from the electronic health record (EHR) system (Epic). Patient records were included if they presented to the ED from January 8, 2018, to June 24, 2022, and were seen by a medical student, as defined by their chart containing a note written and signed by a medical student or a student assigning themselves to the patient's treatment team using the EHR. The chief complaint was used to identify the nature of the patient encounters.^15^, ^16^, ^17^ Each complaint was categorized into one of 20 content domains from the American Board of Emergency Medicine (ABEM) Model of Clinical Practice using an expert consensus model.^16^

### Data analysis

Data elements extracted from the patient encounters of each student included: total number of patients seen, Emergency Severity Index (ESI) acuity level, disposition, and chief complaint. Descriptive analyses were conducted on each of these elements. Student exposure to the 10 core complaints outlined by the Clerkship Directors in Emergency Medicine (CDEM) M3 curriculum^12^, ^18^ was also summarized. Categories of chief complaints in our data set were collapsed by two evaluators when appropriate to best capture exposure to the core presentations.^16^, ^17^, ^19^ Chest pain, abdominal pain, shortness of breath, and altered mental status are defined by CDEM as “must‐know” chief complaints.^12^, ^18^ Exposure to must‐know complaints, was also summarized. All analyses were performed using Microsoft Excel. This project was deemed exempt quality improvement by the University of Wisconsin Health Sciences Institutional Review Board.

## RESULTS

### Overall clinical encounters

A total of 2994 patients were seen across 248 medical students, made up of 4.25 student cohorts. The partial representation of one cohort was due to interruptions during the pandemic. The median number of total ED patients seen by each second‐ or third‐year student was 12.0 (range 1–32).

### Clinical acuity

Student encounters were predominately patients that were emergent (ESI Level 2 or 3), which accounted for 89.4% of all patient encounters (*n* = 2676). Only 2.4% (*n* = 6, range 0–3) of students encountered the highest acuity (ESI 1) patients. Only 22.2% (*n* = 55) encountered a patient who was admitted to the intensive care unit/intermediate care unit.

### Chief complaints

Most students encountered the chief complaints of abdominal pain and chest pain (72.2%, *n* = 179; and 54.8%, *n* = 136, respectively). All other presentations were each encountered by less than 40% of students.

### ABEM content domains

When patient encounters were grouped by ABEM content domain, abdominal and gastrointestinal, cardiovascular, nervous system, and nontraumatic musculoskeletal disorders were encountered most frequently and seen by 81.9% (*n* = 203), 66.1% (*n* = 164), 60.9% (*n* = 151), and 58.1% (*n* = 144), of all students, respectively. Immune system, obstetrics and gynecology, hematologic, and environmental disorders were the least frequently encountered, with less than 10% of students seeing these conditions (Table 1).

**TABLE 1:** Second‐ and third‐year medical student encounters by ABEM Model Of Clinical Practice domains in the emergency department.

Chief complaint domain	Total number of times encountered across all students	Mean number of times encountered	Median number of times encountered	Range	SD	Percent of all students encountering domain (*n*)	Percent of total patients across all students
Abdominal and gastrointestinal disorders	741	3.0	2.0	0–13	2.6	81.9 (203)	24.7
Cardiovascular disorders	407	1.6	2.0	0–7	1.6	66.1 (164)	13.6
Nervous system disorders	314	1.3	1.0	0–10	1.4	60.9 (151)	10.5
Musculoskeletal disorders (nontraumatic)	312	1.3	1.0	0–6	1.4	58.1 (144)	10.4
Thoracic–respiratory disorders	219	0.9	0.0	0–6	1.2	46.0 (114)	7.3
Traumatic disorders	213	0.9	0.0	0–10	1.2	44.4 (110)	7.1
Systemic infectious disorders	127	0.5	0.0	0–4	0.9	31.5 (78)	4.2
Signs, symptoms, and presentations	124	0.5	0.0	0–5	1.0	27.8 (69)	4.1
Head, ear, eye, nose, and throat disorders	103	0.4	0.0	0–4	0.7	29.8 (74)	3.4
Other components	93	0.4	0.0	0–4	0.8	23.4 (58)	3.1
Psychobehavioral disorders	70	0.3	0.0	0–4	0.7	19.0 (47)	2.3
Renal and urogenital disorders	69	0.3	0.0	0–4	0.6	19.8 (49)	2.3
Endocrine, metabolic, and nutritional disorders	42	0.2	0.0	0–4	0.6	9.7 (24)	1.4
Cutaneous disorders	40	0.2	0.0	0–2	0.5	11.7 (29)	1.3
Toxicologic disorders	35	0.1	0.0	0–2	0.4	11.3 (28)	1.2
Immune system disorders	26	0.1	0.0	0–2	0.4	8.1 (20)	0.9
Procedures and skills	19	0.1	0.0	0–3	0.4	5.6 (14)	0.6
Obstetrics and gynecology	14	0.1	0.0	0–2	0.3	4.4 (11)	0.5
Hematologic disorders	13	0.1	0.0	0–5	0.4	2.0 (5)	0.4
Environmental disorders	11	0.0	0.0	0–2	0.3	3.2 (8)	0.4

Abbreviation: ABEM, American Board of Emergency Medicine.

### CDEM M3 curriculum

No students saw all 10 recommended emergency core complaints. The median number of core complaints seen by each student was 3.0 (range 0–7). Most students (87.5%, *n* = 217) encountered less than half of the recommended complaints. Abdominal pain and chest pain were the most frequently encountered, with 79.8% and 54.8% of students encountering the complaints, respectively. Gastrointestinal bleed, pelvic pain/vaginal bleeding, and cardiac arrest were the least frequently encountered, with less than 10% of students seeing these conditions (*n* = 14, *n* = 10, *n* = 1, respectively). A total of 33.1% (*n* = 82) of students saw less than half of the four must‐know presentations. Only 6.9% of students (*n* = 17) saw all must‐know complaints.

## DISCUSSION

This study indicates that there is great variability in the number and type of patients that second‐ and third‐year medical students see in the ED, and despite identical rotations, exposure during EM clinical experiences is far from standardized. The range of number of patients encountered by junior students was 1–32, signifying an appreciable degree of variance regarding patient volume seen between students. Similarly, variation in clinical experience between residents has also been described, where the range corresponds with about 1 year of EM training.^19^ In residency training, this degree of variance in volume of patients encountered could have implications for clinical competency^20^; the same may be true for learners at the undergraduate levels of medical education.

Junior medical students see a limited number of high‐acuity patients as defined by those requiring admission to the intensive care unit or categorized as critical ESI Level 1 acuity. The CDEM curriculum defines the ability to understand and describe the clinical approach to an unstable patient as must‐know content.^12^ Additionally, a main objective for the CDEM M3 curriculum is to develop a gestalt for identifying “stable versus unstable.”^12^ If students are not encountering enough “sick” or “unstable” patients, this may delay their ability to develop this skill and gestalt. This idea that the ability to distinguish “sick” from “not sick” is poorly taught in medical school has been previously outlined in the literature.^21^

Abdominal pain, chest pain, and dyspnea were the most frequently encountered, similar to the experiences of fourth years.^22^, ^23^ No medical students encountered all 10 core presentations recommended by the CDEM curriculum; only 6.9% of students saw all four must‐know complaints. This parallels the findings of previous research showing that fourth‐year students do not encounter all recommended conditions.^22^, ^23^, ^24^ Cardiac arrest was the least encountered, with only one student encountering this complaint. This was also consistent with the experiences of fourth‐years.^22^, ^23^

Prior research has implemented the use of clinical logs, “passports,” or “e‐portfolios” as a means of tracking students' clinical experiences, in an attempt to identify gaps and ultimately standardize clinical experiences.^4^, ^5^, ^8^, ^10^, ^22^, ^24^, ^25^, ^26^, ^27^, ^28^ This study provides further support for tracking patient encounters as described in the literature.

## LIMITATIONS

This study has several limitations. First, this study was conducted at a single site. Additionally, it is likely that students interacted with patients not reflected in the EHR because they either did not assign themselves to the patient or did not complete a note. Furthermore, the CDEM curriculum was designed for a 4‐week rotation^13^, ^19^; however, this study is of a 2‐week rotation. Given that the majority of EM M3 clerkships are 2 or 4 weeks,^4^, ^19^ there may be a need for a curriculum that takes into consideration 2‐week rotations. Finally, a limitation is the subjective nature of the categorization of chief complaints. Other studies have also highlighted that the “lack of standardization of terminology”^23^ in the CDEM guidelines. Core complaints may be inconsistently categorized, and the chief complaint does not always reflect the workup performed. Altered mental status, for example, may be due to polysubstance abuse and receive a toxicologic rather than a neurologic workup. However, in EM, the final diagnosis is not known at the outset of the case, so a chief complaint‐based approach was deemed to have the greatest fidelity for examining clinical exposures.

## CONCLUSIONS

There is significant variability in the patient presentations encountered by second‐ and third‐year medical students in the ED with wide ranges of patient volume and presentations encountered. These early students did not encounter the Clerkship Directors in Emergency Medicine–recommended core presentations for M3 students.

## AUTHOR CONTRIBUTIONS

Ines Hoxha: study concept and design, analysis and interpretation of the data, drafting of the manuscript, critical revision of the manuscript for important intellectual content. Daniel J. Hekman: acquisition of the data, statistical expertise. Benjamin Schnapp: study concept and design, analysis and interpretation of the data, critical revision of the manuscript for important intellectual content, acquisition of funding, study supervision.

## CONFLICT OF INTEREST STATEMENT

The authors declare no conflicts of interest.
